# Individual preferences for scented water bowls in dogs

**DOI:** 10.3389/fvets.2025.1688084

**Published:** 2025-10-13

**Authors:** Rituparna Sonowal, Nathaniel J. Hall, Anastasia C. Stellato

**Affiliations:** Department of Animal and Food Sciences, Texas Tech University, Lubbock, TX, United States

**Keywords:** hydration, water consumption, scented sleeves, preference, health

## Abstract

**Introduction:**

While food preferences have been extensively studied, much less is known about water preferences in dogs, especially regarding preferences for non-consumptive scented items attached to water bowls. As a form of sensory enrichment, scents can increase engagement and were used here to assess whether dogs show individual scent preferences when drinking water. This research explores whether individual preferences for non-consumptive scented sleeves on the water bowl influence dogs’ water consumption, considering that adequate hydration is vital to their health and physiological functions. Establishing individual preference for such items may promote hydration in dogs, which could support maintaining hydration levels for dogs, especially those with existing health conditions.

**Methodology:**

Experiment 1 evaluated the water consumption levels of dogs (*N* = 20) in household settings over 4 days using four bowls with sleeves (three scented and one non-scented). Each bowl was placed on a custom-built scale to record daily water consumption (mL/kg) to establish the individual bowl preference. Experiment 2 recruited dogs (*N* = 10) from Experiment 1 to record water consumption over 14 days using two bowls embedded with sleeves preferred scented emulsified sleeves (based on Experiment 1; chicken or beef, and non-scented). Owners completed a brief survey to report their dog’s diet type, daily physical activity levels (<30 min, >30 min – 1 h, >1 h), method of feeding (free-fed or scheduled), and dog age.

**Results:**

In Experiment 1, there was no single scent that was preferred across dogs (*p* = 0.15). In Experiment 2, dogs had greater water consumption with their preferred emulsified scented sleeves compared to the non-scented (*p* = 0.02). Increased water consumption was associated with dry diet (*p* = 0.02) and most water consumption occurred during the evening (*p* < 0.001; vs. afternoon). Age and daily physical activity levels did not influence water consumption in dogs.

**Discussion:**

Findings suggest that using emulsified scented sleeves is associated with water consumption preference in pet dogs, and their preference for a scented sleeve over a non-scented one was sustained across experiments and through the 14-day data collection period. Thus, dogs prefer to consume water from water bowls with scented sleeves, which may be helpful with hydration and should be investigated in future work.

## 1 Introduction

Water is essential for maintaining the health of companion animals and should be readily available to support hydration and a range of physiological functions, including the regulation of body temperature and the removal of metabolic waste (1). Water loss in dogs naturally occurs through mechanisms such as thermolysis, urinary excretion, salivation, and respiratory evaporation (2), with a thirst response typically evoked after 0.5–1% loss of body weight (3, 4). For dogs, inadequate water consumption is associated with reduced body weight, decreased urine output, increased concentrations of sodium and other substances in the blood and urine, and greater susceptibility to heat stress and risk of heat stroke (5–8). When prolonged or severe, dehydration can also result in kidney failure and organ dysfunction (9, 10). Therefore, promoting and maintaining adequate hydration in dogs is important for overall health and reducing the incidence and severity of disease. Despite the importance, there is no agreement on the optimal water intake volume for dogs.

With aging, water-related metabolism can be altered due to factors like decreased thirst perception, and this could lead to reduced hydration (11). Dogs’ recommended water intake is typically estimated to be 40–60 mL/kg/day (12) and can be reported as mL/kg of body weight, mL/kg of dry matter intake, or mL/kcal of metabolizable energy (13). The National Research Council (2006) recommended that the water-to-calorie intake ratio should be 1.0:1.0 mL/kcal of metabolizable energy, though this has been suggested to be an underestimation of another proposed range of 1.2:1.0–1.4:1.0 mL/kcal (14).

Several factors are known to influence water intake, such as diet composition. Water consumption in dogs has a reported linear relationship with food intake and commonly occurs post-meal, with diet composition (salt and moisture content) playing a major influence on hydration status (15). Dogs can adjust their water intake accordingly, with dry or high-salt diets increasing both total volume and duration of water consumption (15). For instance, dogs that eat canned wet food typically consume 24.2 mL/kg a day (2), whereas those that eat dry food consume 62.2–73 mL/kg (2, 14, 16, 17).

Recent studies on working dogs and experiments performed on dogs at laboratories have explored ways to promote water intake and improve hydration in dogs. These studies that observed that nutrient-enriched water (14, 18), chicken-flavored water (19), flavored electrolyte solution (20), and cold water (21) have resulted in greater water consumption. These interventions have also been associated with additional benefits, such as improved maintenance of body temperature after exercise in working dogs (22); however, this area of research is limited and needs to be further explored.

To date, most of the studies have explored the addition of consumptive additives in water to promote water intake in dogs. However, no studies have explored preferences for non-consumptive items and whether such preferences can influence water consumption in dogs. These non-consumptive interventions could be accessible and easy for pet owners to apply in their homes. Given that dogs show a natural preference for food-related scents (23, 24), one potential strategy to promote water consumption involves integrating food scents to the water bowl by adding a food-scented sleeve around the bowl. Prior research indicates dogs demonstrate increased engagement with toys with individual preferred scents compared to non-scented or non-preferred scents, suggesting that individual preferences for scented sleeves may effectively encourage water consumption in owned dogs (24, 25).

The current study aims to explore whether dogs show an individual preference for a water bowl with an added scented sleeve. The sleeves were made of silicone and fit tightly under and around the external surface of the bowl. The sleeves were infused with natural food-derived scents (beef, peanut butter, and chicken). Their purpose was to attract dogs through olfactory cues, thereby increasing their approach the bowls and potentially improve water consumption. To do this, we conducted two experiments. Experiment 1 aimed to evaluate dogs’ scent preference based on total water consumption across three scented and one non-scented water bowl sleeve. Experiment 2 aimed to assess if bowls with preferentially scented sleeves, in comparison with non-scented sleeves, influence daily water consumption over a 14-day period. Also, Experiment 2 aimed to determine if daily water consumption varies according to diet composition (wet food or dry food). The influence of other factors, such as age, daily physical activity levels, and time of day was also assessed. We hypothesized that dogs would have a preference for drinking from the bowl with their preferred scented sleeve compared to a non-scented sleeve.

## 2 Materials and methods

The study protocol was reviewed and approved by the Institutional Animal Care and Use Committee of Texas Tech University (AUP# 2023-1389).

### 2.1 Experiment 1: assessing the water consumption levels between scented and non-scented sleeves

#### 2.1.1 Subjects

Dogs (*N* = 20) were recruited using social media and online advertising platforms, where dog owners were provided with informed consent and were asked to complete a brief survey to determine their dog’s eligibility. This online survey was created on Qualtrics® and distributed to collect dogs’ demographic information (i.e., age, sex, weight, breed, health status). To be eligible for participation, dogs needed to be ≥ 1 year of age, without any current health issues, up to date on vaccinations, and residing near the study site.

#### 2.1.2 Methodology

All testing took place in the homes of consenting dog owners. To minimize for potential bias in multi-dog households, the study primarily recruited single-dog households. However, six dogs that were from three multi-dog households, two from each household, were included under specific criteria that the dogs did not share their water bowls, and testing was structured to control for potential cross-interference. In two of the multi-dog households, both dogs were tested simultaneously, while in the third, they were tested at separate times. For the latter, a barrier was installed in the kitchen to prevent the smaller dog from accessing the experimental bowls intended for the medium-sized dog, reducing the risk of bias during drinking events.

**Figure 1:**
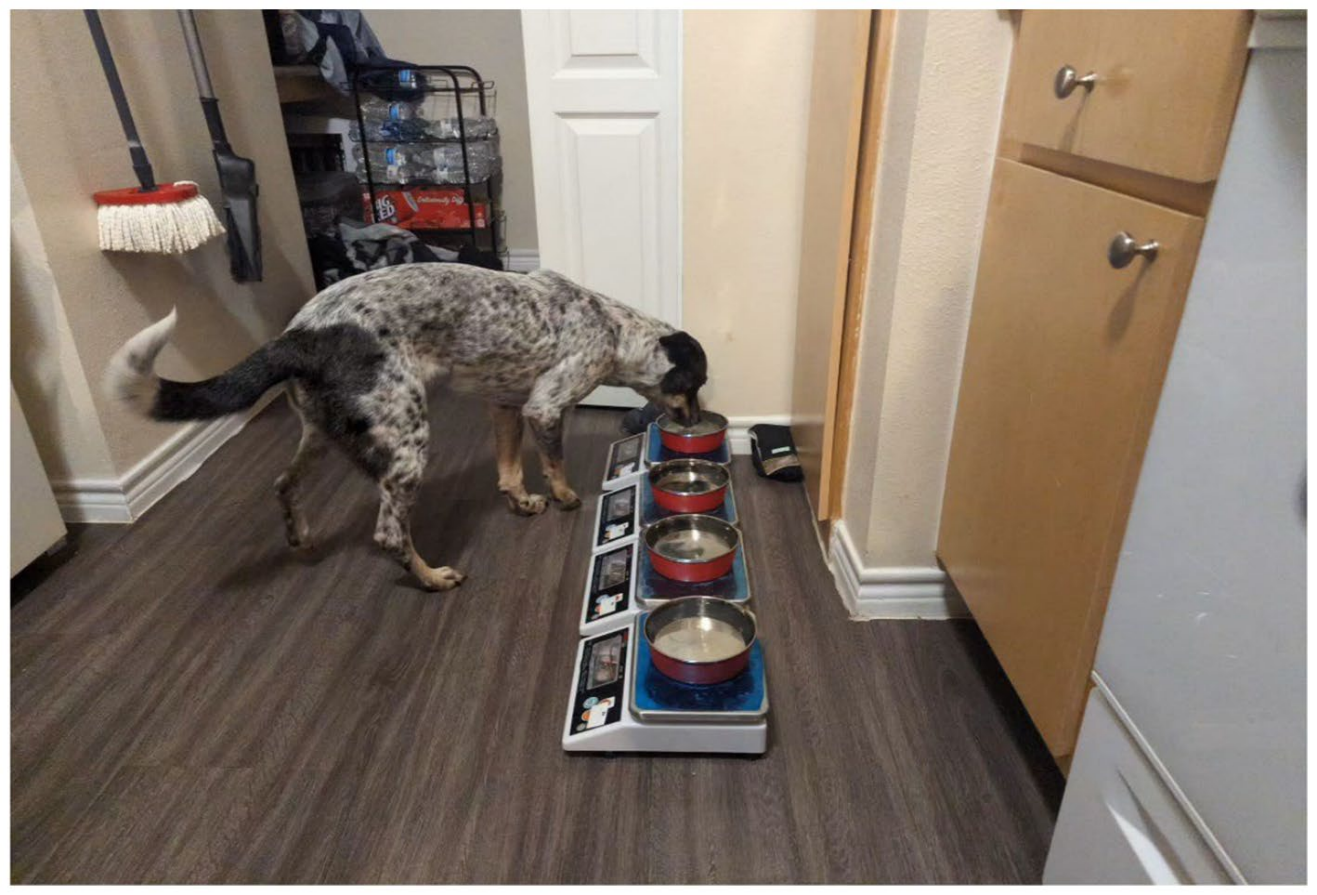
Dog participant (Willow) consuming water from the lineup of the water bowls. Four bowls identical in appearance and size, embedded with different scents, were simultaneously presented to the test dog on custom-built scales placed 2 inches apart from each other.

Each participating pet dog was provided with four water bowls (4.6″ wide, 2.3″ tall) each embedded with either a scented or non-scented sleeve (beef, chicken, peanut butter, and non-scented; Playology®). All scented sleeves were red in color and owners were blinded to the scent types. Sleeve identity was known only to the researchers, marked discretely beneath each bowl. Each bowl was placed on top of a custom-built scale. The bowls were placed 2″ apart and located either in the dog’s usual water bowl location or another easily accessible area, as determined by the owner (Figure 1). All bowls were filled with tap water or purified water, based on the owner’s preference, up to the brink of each bowl. All bowls were always simultaneously available. Free access to the water bowls depended on the dog’s typical routine; some dogs had continuous free access to the water bowls, while a few dogs (*N* = 3) were in a crate for a portion of the day.

**Figure 2:**
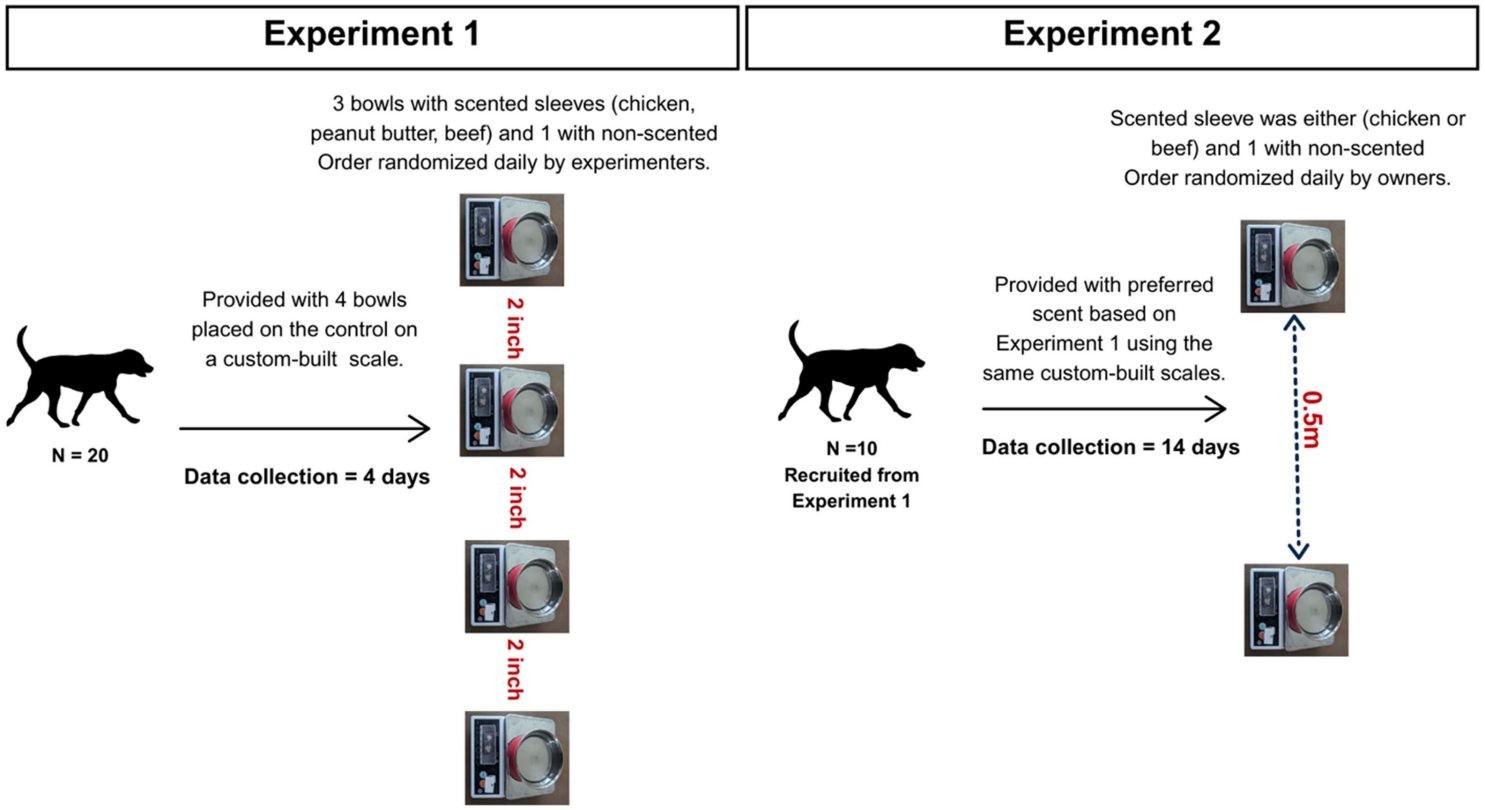
A schematic diagram of the study design.

Prior to testing, all existing water bowls were removed for the study duration. The test was conducted for four consecutive days. The owners were instructed to refill the bowls even if a single bowl was partially empty, without lifting the bowls from the measuring scales. In addition, if, by any chance, bowls were toppled over by dogs, owners were instructed to return them to the same measuring scale. Once every 24 h, the experimenter visited the household to refill and clean bowls (e.g., remove any food debris or upon owner request), and randomly rearranged the positions of the bowls to account for order effects. At the end of the fourth day, the bowls were collected, and the owners were compensated for their participation. A schematic diagram detailing the study design of Experiments 1 and 2 is provided in Figure 2.

**Figure 3:**
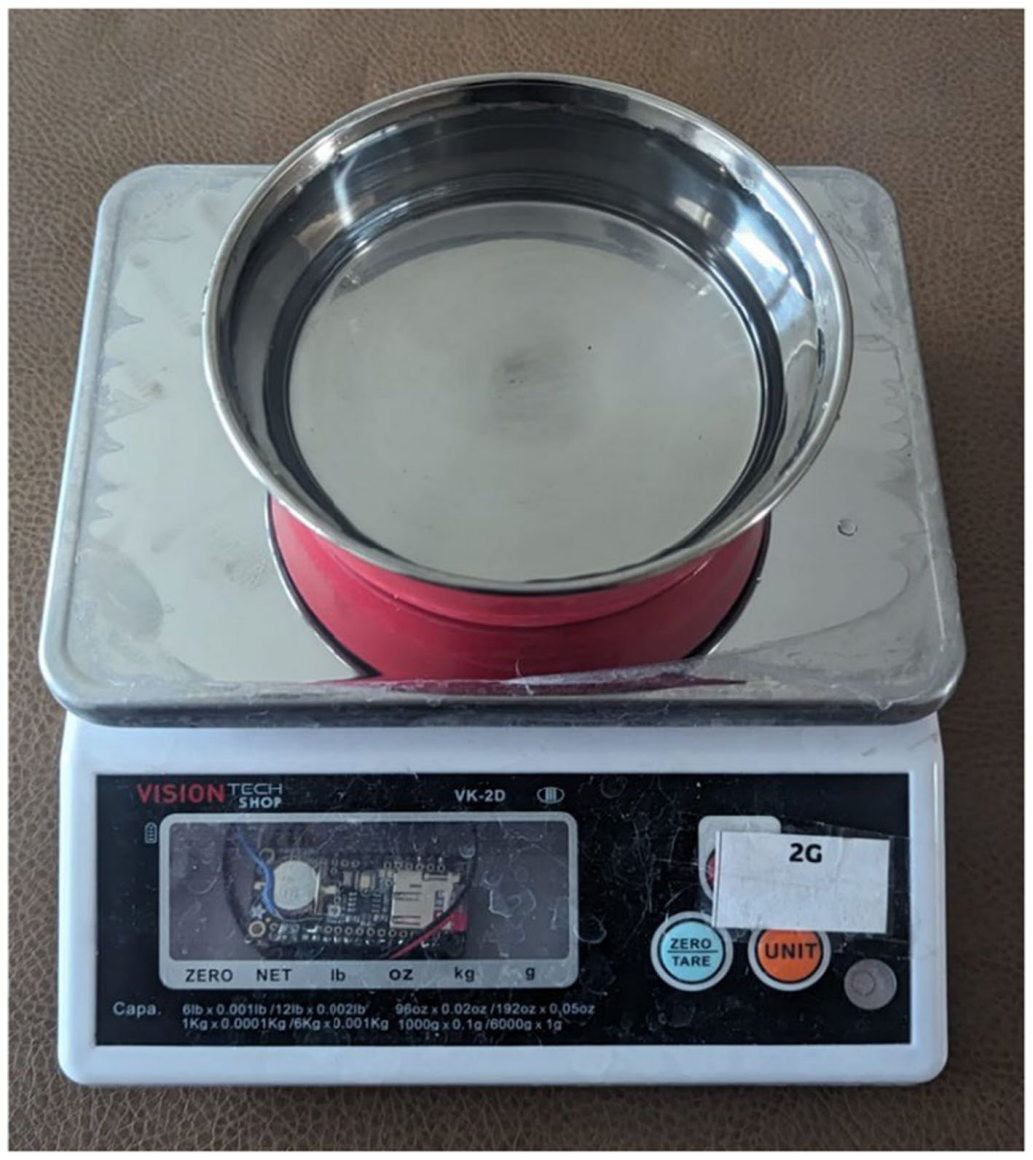
Displaying a custom-built scale that had an accuracy of 1 g and recorded weights every 5 s in Experiment 1 and every 30 s in Experiment 2. Scales were calibrated using a 2,000 g calibration weight and regression coefficients were applied to each scale to ensure precision in measurements.

To record the water consumption of dogs, 16 custom digital food weighing scales were designed and built. To distinguish the scale, each scale had a number and a letter on top of it. Each scale was controlled by an Adafruit Feather 32u4 Bluefruit and Adafruit Feather 32u4 Adalogger along with a load cell amplifier. The Adafruit Feather 32u4 Adalogger acts as a data logger with a built-in micro-SD card socket for data storage and battery-backed real-time clock (Figure 3). The SparkFun Load Cell Amplifier-HX711 was connected to the load cell of a VK-2D Kitchen Scale Series. The apparatus was powered by an external lithium-ion 4,400 mAh battery. The scale was programmed to record timestamped weights within a 1 g accuracy every 5 s. Prior to acquiring weight measurements, each scale underwent a calibration process. A precision 2000 g calibration weight was added and removed from the scale. The regression coefficients for the scale were then calculated, and accuracy was confirmed using multiple smaller weights to be within 1 g accuracy. Scales were calibrated before each testing session.

Weight samples were measured to record continuous water consumption every 5 s for 24 h for four different water bowl sleeves (beef, peanut butter, chicken, and non-scented). To identify water consumption events, the raw weight CSV data file was processed with a custom-built algorithm for R in R Studio. We first calculated a 5-point moving average of the weights from the raw data file using the R function movavg. We filtered out any raw weight values that deviated from the calculated moving average by more than 5 g. These were indicative of unstable weight readings and were thus filtered out to attain reliable weight data representation. Then the filtered data set of stable weights was passed to an algorithm named “water consumption” to calculate serial losses of weight (i.e., water). An event of water consumption was only considered when there was a weight loss of more than 2 g within subsequent time points. All weight losses greater than 2 g were retained to indicate water consumption events. The algorithm can be found in the data availability section.

To validate the algorithm, it was applied to 12 data files (~288 h of data collection) to measure the water consumption events and manually calculate all weight losses based on visual inspection of the raw data. The values obtained from the visual inspection of the raw data files, and the algorithm approach were then compared according to the timestamp. Based on this assessment, we detected a miscalculation in five out of 95 identified water consumption events. These miscalculations from the raw data were believed to be due to the transient fluctuations in the weight for reasons other than drinking (e.g., a dog nudging the scale or putting some pressure on the scale). The fluctuations were identified when the weight first increased to a value and then returned to its original stable weight value. The fluctuations that occurred within 10 s of the time frame and then returned to their original value of the stable weight after 10 s were considered transient fluctuations in our study. In total, across the 288 h of data collection, 22.24 g of water were determined to be miscalculated by the algorithm out of a total of 3941.44 g of water consumption calculated. This reflected an overall 0.56% error rate, which was determined sufficient to evaluate 24-h water consumption from each bowl.

### 2.2 Experiment 2: assessing the water consumption of owned dogs using emulsified scented sleeve and non-scented sleeve

#### 2.2.1 Subjects

Among the dogs (*N* = 20) in Experiment 1, there was a trend for greater water consumption from bowls with chicken scented sleeves. Some dogs showed preferences for chicken (*N* = 9) and beef (*N* = 7) compared to other scents, as indicated by volume of water consumed. On the basis of these findings, we sampled dogs (*N* = 10) from Experiment 1 using stratified random sampling to explore whether individual preferences for a scent influence water consumption. Dogs were divided into two strata according to their preferred scented sleeve, chicken or beef, and then randomly sampled within each group. Of 10 selected dogs, five preferred the beef scent and five preferred chicken scent.

#### 2.2.2 Methodology

Previous participants were contacted via email and provided with informed consent through a brief online survey in Qualtrics®. The survey outlined participation involvement and included additional questions about their dog, including diet composition (Dry only, Wet only, or a combination), daily physical activity levels (<30 min, 30 min – 1 h, >1 h), and feeding schedule (free-fed, or the number scheduled meals per day).

Similar to Experiment 1, all testing took place in the homes of consenting dog owners. Of the 10 participating dogs, four lived in multi-dog households, with the eligibility criteria that dogs did not share water bowls. In each of the four multi-dog households, dog bowls were separated using a barrier or placed in different rooms, ensuring no cross-over influencing the data. Data was recorded for 24 h each day for a period of 14 days, and all scales were calibrated prior to use.

Each dog was provided with two water bowls, one fitted with either an emulsified scented sleeve containing their preferred scent (identified in Experiment 1) and the other with a non-scented sleeve (Playology®). The emulsified scented sleeve, developed by Playology®, were formulated to retain scent longer and deliver a stronger olfactory signal compared to the non-emulsified sleeves used in Experiment 1. These sleeves were chosen to better reflect the product’s intended market use and to allow assessment of how individual scent preference influences water consumption.

Bowls were placed within 0.5 meters of each other, in locations that were simultaneously accessible, with access depending on the dog’s routine. In Experiment 2, a few dogs (*N* = 3) were kenneled for part of the day. Each bowl was filled to the brim with water, and the same custom-built scales from Experiment 1 were used to record water consumption. Prior to testing, all existing water bowls were removed. To better capture water consumption, the testing period was extended from 4 to 14 days. Owners were instructed to refill the bowls as needed and to switch the location of the bowls (including their respective scale) daily at the same time to account for potential side bias. To verify compliance with instructions, researcher provided owners with a sheet to record if they changed the bowl position. Every fourth day, the experimenter visited the household to change the batteries, check the data, and adjust the location of the bowls, if needed. Owners were instructed to notify the researcher if they no longer observed the red indicator light on the scale, so that the researcher could come and change the external battery prior to the next scheduled visit. To accommodate the longer testing duration and conserve battery life, the algorithm used in Experiment 1 was refined to record water consumption every 30 s instead of every 5 s. At the end of the testing session, measuring scales and bowls were collected, and owners were compensated for their participation.

### 2.3 Statistical analysis

Data across both experiments were downloaded from the SD card present in custom-built digital food weighing scales. The raw data was processed by the developed algorithm in R studio to identify all water consumption events. The daily water consumption was calculated for each dog for each scent. This was then divided by the dog’s body mass (kg) to calculate water consumption per kg of body mass (mL/kg) (26). All statistical tests for both experiments were conducted in R Studio. The models were fit using the *lme4* package (27). The *lmerTest* package was used to calculate the respective *p*-values for each model (28). Error bar plots were created using the ggplot2 package (29).

For Experiment 1, to explore the influence of scent on water consumption, a linear mixed-effect model was used to compare daily water consumption predicted by scent (beef, peanut butter, chicken, no-scent) with a random intercept of the individual. For Experiment 2, the raw data were processed to calculate water consumption per kg of body mass (mL/kg/day). We also calculated water consumption by the time of day for time between 6 a.m. to <12 p.m. (Morning), 12 p.m. to <6 p.m. (Afternoon), 6 p.m. to <12 a.m. (Evening), and 12 a.m. to <6 a.m. (Night).

Two linear mixed-effect models were created to explore the influence of sleeve type (preferred scented vs. non-scented), diet composition (dry vs. wet), daily physical activity levels, and time of the day (morning, afternoon, evening, and night) on daily water consumption, with dog included as a random effect. Due to multicollinearity with diet composition and physical activity, the influence of age was evaluated in a separate univariate model. Additionally, two linear mixed models were created to explore whether the number of water consumption events per day and water quantity consumed per event were predicted by sleeve type (preferred scent vs. non-scented), with the dog included as a random effect. Feeding schedule could not be assessed in this study due to limited variation in feeding times.

## 3 Results

### 3.1 Experiment 1

#### 3.1.1 Descriptives

In Experiment 1, 20 dogs (12 females and 8 males) were analyzed. Dog age ranged from 1 to 14 years, with a mean age of 5.4 years. A total of 12 dog breeds were represented: Mixed (7; 5 medium/large, 2 small), Bulldog (2), Poodle miniature (2), Australian cattle dog (1), Border Collie (1), Chihuahua (1), Welsh Corgi (1), Doodle (1), French bulldog (1), Jack Russell Terrier (1), Old English Sheepdog (1) and Golden Retriever (1). Ten were small and 10 were large size dogs.

**Figure 4:**
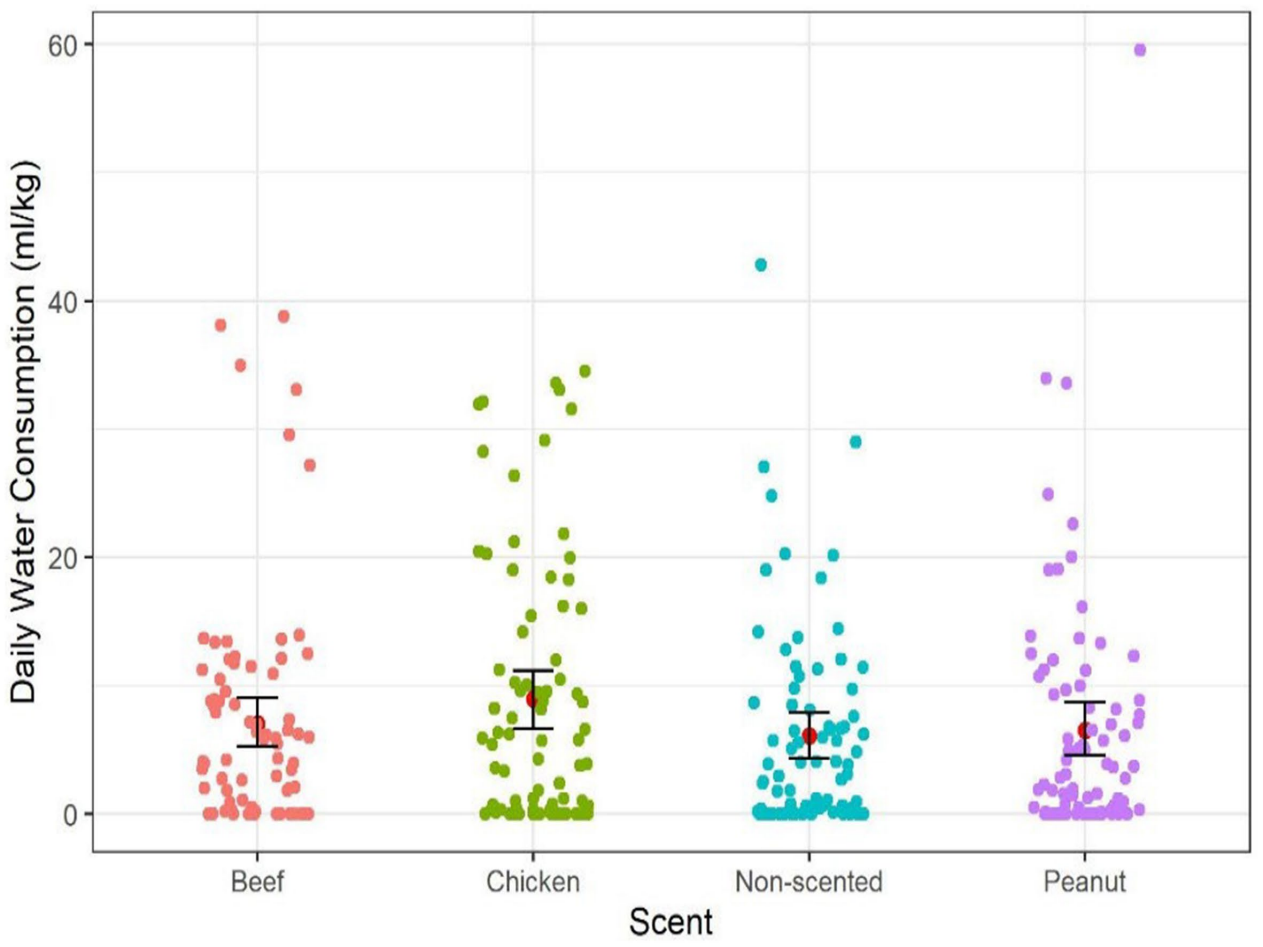
Daily water consumption (ml/kg) from each bowl, three scented (chicken, beef, and peanut-butter) and one non-scented sleeve across dogs. The red dot represents the mean, and error bars show the non-parametric bootstrap estimating the 95% confidence interval.

**Figure 5:**
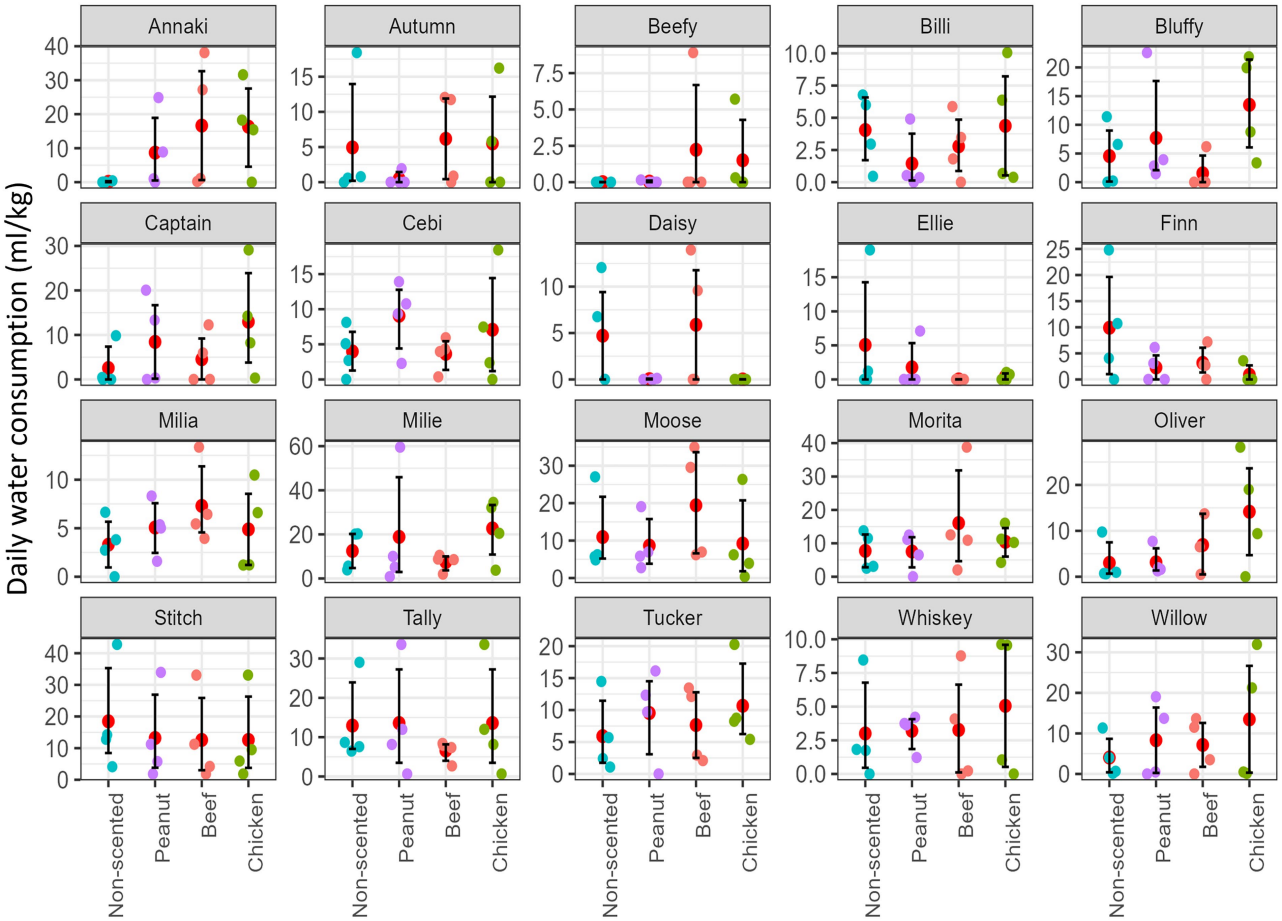
Daily water consumption (ml/kg) for each dog (*N* = 20) from scented sleeves (chicken, beef and peanut-butter) and non-scented sleeves bowls. The red dot represents the mean and error bars show estimated the 95% confidence interval using a non-parametric bootstrapping method. Colored dots represent individual daily consumption measures over the 4 days of data collection.

In Experiment 1, a total of 1,122 water consumption events were recorded over 1,920 recorded hours of observation from 20 dogs. Dogs consumed on average (±SE) of 28.6 (±4.70) ml/kg per day. Dogs consumed on average (±SE) of 8.97 (±2.28) ml/kg from the chicken scented sleeved bowl, 7.05 mL/kg from beef (±2.00), 6.55 mL/kg from peanut butter (±2.15), and 6.08 (±1.78) ml/kg from the non-scented sleeved bowl per day on average (Figure 4). Individual variability in water consumption preference was observed across dogs, with some dogs consuming more water from the scented sleeves, whereas others consumed more from the non-scented ones (Figure 5).

#### 3.1.2 Daily water consumption for consumption (ml/kg) between scented and non-scented sleeves

Results from the linear mixed regression model reveal no difference in daily water consumption between scented sleeves (*χ*^2^ = 5.26, *df* = 3, *p* = 0.15). *Post hoc* tests indicate a non-significant trend of greater water consumption from chicken-scented sleeve compared to the non-scented (*t* = 2.13, *p* = 0.089).

### 3.2 Experiment 2

#### 3.2.1 Descriptive data

A total of 10 dogs (7 females and 3 males) were analyzed. Dog age ranged from 2 to 14 years, with a mean age of 6.5 years. Four small breeds were represented: Bulldog (1), Poodle miniature (1), Welsh Corgi (1), Jack Russell Terrier (1), and four large breeds Australian Cattle Dog (1), Border Collie (1), Old English Sheepdog (1) and Mixed (3; 3 medium/large) were tested.

**Figure 6:**
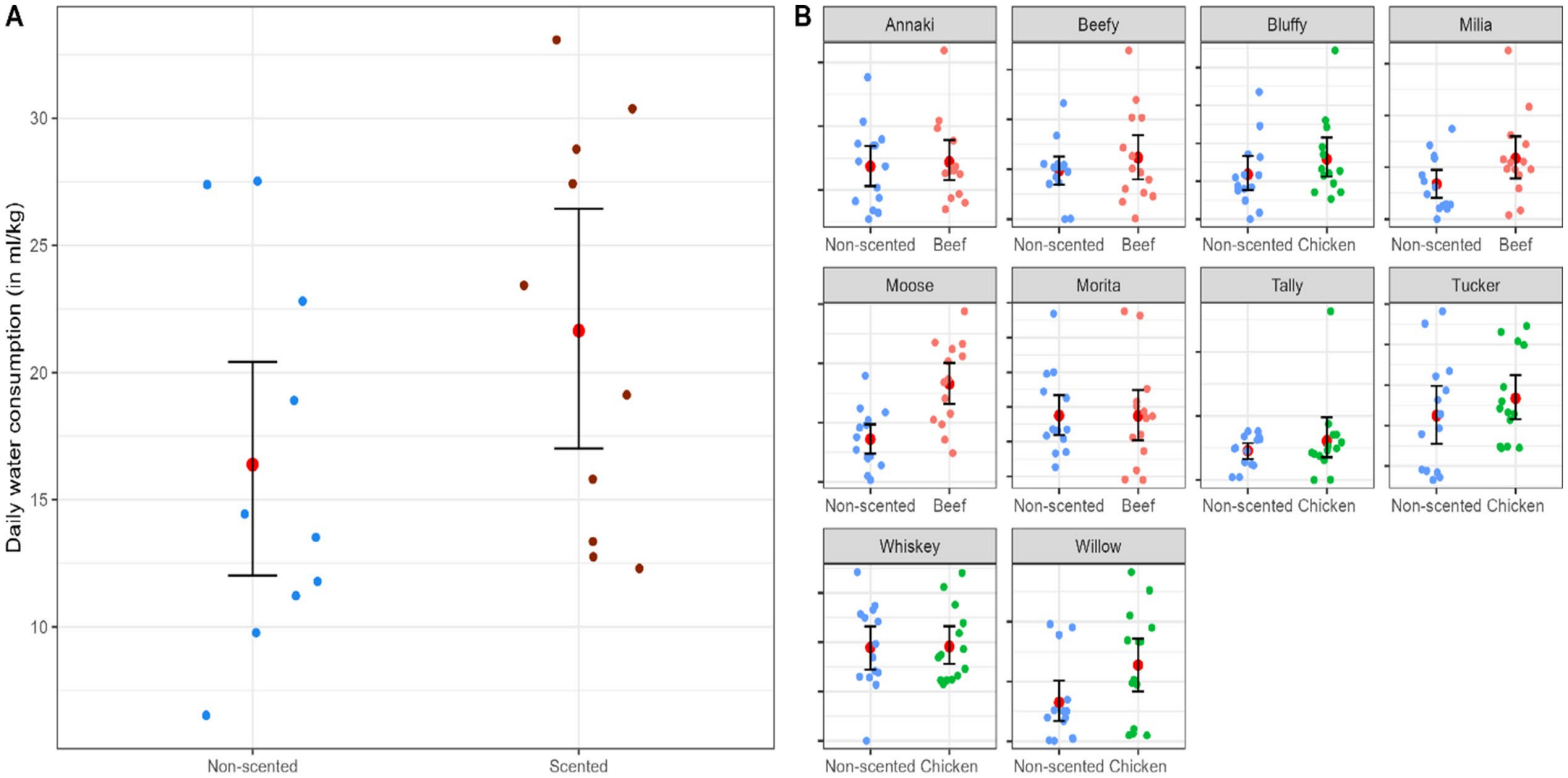
The daily water consumption (ml/kg) of dogs (*N* = 10) from bowls with the preferred scented and non-scented sleeves, displaying more water consumption occurring with the preferred scented sleeve. The red dot represents the mean, and the error bar represents the 95% confidence interval computed using a non-parametric bootstrapping method. Each dot represents the individual daily consumption measures over the 14 days of data collection. **(A)** The average daily water consumption for their preferred scented and non-scented sleeves across dogs. **(B)** Each dog’s average daily water consumption for each sleeve type (chicken, beef, and non-scented).

The average (±SE) daily water consumption was calculated from the summed total water consumption across all sleeves. The average (±SE) daily water consumption from their preferred scented sleeve was 21.64 mL/kg (±2.52), and 16.40 mL/kg (±2.34) from the non-scented sleeve (Figure 6). Across all dogs, the average (±SE) daily water consumption in the morning was 8.06 (±2.04) ml/kg, then increased to 9.02 (±1.44) ml/kg in the afternoon and peaked at 17.8 (±2.26) ml/kg during evening, before reducing to 3.17 (±1.54) ml/kg at night. Dogs that were fed dry food had an average daily water consumption of (44.5 ± 4.43 mL/kg), and those fed a combination of wet and dry food was (23 ± 2.45 mL/kg). For the feeding schedule, three owners reported feeding their dogs a free-fed meal, and seven owners reported feeding their dogs at scheduled times (morning and evening), except one who reported their dog fed only during the evening.

#### 3.2.2 Daily water consumption (ml/kg) between emulsified scented and non-scented sleeves

Results from the linear mixed regression models indicate that dogs’ daily water consumption varied by sleeve type (*p* = 0.02; Table 1), with dogs having an increase of 5.26 mL/kg water consumption from the water bowl with their preferred scented sleeve in comparison to the non-scented sleeve (Figure 6). Dog’s daily water consumption varied by diet composition, with dogs having statistically greater water consumption that were fed dry food as compared to a combination of wet or dry food (*p* = 0.02, Table 1). Dogs daily water consumption significantly varied by time of the day (Table 1), with highest level of water consumption occurring in the evening (*p* < 0.001; vs. afternoon) and lowest at night (*p =* 0.02; vs. afternoon). Daily water consumption was not influenced by daily physical activity levels (>1 h vs. < 30 min, *p* = 0.24; >30 min – 1 h, *p* = 0.41) nor dog age (*p* = 0.56; Table 1).

**Table 1:** Mixed linear regression models displaying estimates, 95% CI and p-value that assess the influence of sleeve types (preferred scented and non-scented), diet composition, age, and daily physical activity levels on daily water 329 consumption (ml/kg) with dogs (*N* = 10) included as a random effect.

Variables		Estimates	CI (95%)	*p*-value
Daily water consumption (Model 1)				
Sleeve type	Preferred scent vs. non-scented	5.26	1.54, 8.97	**0.02**
Diet composition	Wet food vs. dry food	−10.75	−17.68, −3.82	**0.02**
Daily water consumption (ml/kg) (Model 2 and Model 3)				
Time of the day	Morning vs. Afternoon	−0.96	−5.44, 3.51	0.68
	Evening vs. Afternoon	8.75	4.27, 13.22	**<0.001**
	Night vs. Afternoon	−6.03	−10.50, −1.50	**0.02**
Physical activity	>1 h vs. <30 min	5.03	−2.11, 12.19	0.24
	>30 min – 1 h vs. <30 min	3.57	−3.77, 10.89	0.41
Age		−0.20	−0.81, 0.44	0.56

Bolded values indicate significance at *p* <0.05.

#### 3.2.3 Daily consumption events and water consumed per event between scented and non-scented sleeves

There were numerically more consumption events per day from bowls with preferred scented sleeves compared to non-scented sleeves, but the effect was not significant (β1 = 3.4, CI: −1.32, 8.09, *p* = 0.16). Also, there was no significant difference in the water consumed per drinking event per day between the preferred scented and non-scented sleeves (β1 = 0.6, CI: −0.16, 1.36, *p* = 0.12).

## 4 Discussion

The current study evaluated the preference for scented sleeves and whether such preferences for non-consumptive additives attached to water bowls can promote water consumption in owned dogs. Based on evaluations of daily water consumption from Experiment 1, results suggest that dogs have individual preferences for scent, as no one scent was deemed on average preferred by participating dogs. There was a non-significant trend toward increased water consumption from bowls with scented sleeves compared to non-scented, with a trend toward chicken scent. To achieve statistical significance, a sample size of 560 dogs would have been required, with 80% power estimation and an effect size of 0.014.

In Experiment 2, a subset of dogs from Experiment 1 were presented with just the scented bowl with the greatest consumption in Experiment 1 for a 14-day (compared to 4 days in Experiment 1) data collection period. Experiment 2 results revealed increased daily water consumption from the preferred scented sleeves compared to non-scented.

Previous studies have demonstrated that scent-based enrichment strategies, particularly when tailored to individual olfactory scent preference, can increase engagement and play behavior in dogs (24, 25). Moreover, the provision of scent has not only been shown to increase exploration but also reduce stress-related behavior in dogs, thereby improving their overall welfare (30). The current study provides further evidence that scent-based enrichment can be a powerful tool that can be applied to enhance engagement with items and promote water consumption in dogs, as dogs prefer to consume from the scented sleeve bowl. Thus, this type of intervention, i.e., adding preferred scented sleeves on the base of the bowl, can make the bowls more appealing to the dogs, potentially increasing water consumption in dogs, thereby benefiting the health and overall welfare of dogs. Although habituation can be a major factor that could influence scent-based preferences (31), dogs in the current study demonstrated a consistent preference and increased uptake from their preferred emulsified scented sleeves throughout the 14-day period, suggesting dogs may have more persistent preferences under these condition. Analysis of drinking behavior revealed that dogs numerically had more drinking bouts with the scented bowl and greater consumption per bout, although this did not reach statistical significance. This suggests that the overall increased water consumption is likely driven by both moderate increases in the number of drinking bouts and consumption per bout.

Diet composition was the greatest contributor to water intake, with dogs fed dry food consuming more (21.5 mL of water/body weight per day) water compared to those whose diets included wet food. This finding aligns with previous studies that suggest dogs housed in laboratory settings can consume approximately 60–73 mL/kg when fed dry food (2, 14) compared to 24 mL/kg of water when fed wet food (2). While those studies focused on dogs in controlled environments and often following periods of water deprivation or in suboptimal hydration state, our study provides insight into hydration levels in pet dogs in household settings.

When observing the daily drinking pattern of dogs, water consumption was highest in the evening and lowest overnight. The observed drinking pattern may reflect their evening physical activity, when most owners are home and/or more available to facilitate it (e.g., walks, active play); however, daily physical activity levels were not associated with water consumption. Three dogs did not have continuous access to water due to brief periods of kennel confinement when family members were not at home, and though this unlikely biased the overall findings, it may have contributed to the trend of increased water consumption in the evening once they were removed from the kennel. It is likely that the current small sample size and low variability between daily physical activity levels reported by owners prevented this relationship from being observed. Age did not influence the water consumption of dogs; however, we had a small sample size and mostly younger dogs. Further research is needed to explore water consumption in aging dogs.

The current study had several limitations. The variability in individual scent preferences prevented the detection of a universal preference across dogs. In general, non-significant findings may reflect the influence of unmeasured factors, such as prior scent exposure, seasonal variation, or ambient temperature, which could affect dogs’ water consumption and preferences. These factors were not captured in the present study but may help explain variability in the results. Future research with larger sample sizes and broader settings will be important to test these influences and assess the generalizability of our findings beyond household dogs. Further, although owners were instructed to refill the bowls as needed, instances may have occurred where the bowls, particularly due to their smaller size, were emptied more frequently by larger dogs. In some cases, owners may not have refilled the bowls promptly due to lack of supervision or being away from home, leading dogs to switch to another bowl simply because the first one was empty, rather than out of true preference. For instance, there were four instances where dogs had no water consumption for a day from the non-scented sleeves. Also, other water consumption events may have taken place outside of the home, such as when taken outside for physical activity. Self-reported daily physical activity levels from owners may be subject to social desirability bias, potentially leading to overestimation of duration or intensity. Increasing the sample size in future studies may help mitigate the effects of such bias.

## 5 Conclusion

In this study, we successfully developed a novel tool to measure daily water consumption in owned dogs within household settings, using an algorithm that automatically detects consumption events with 0.56% error. The study also demonstrated that dogs have individual scent preferences, and these preferences were associated with increased water consumption from bowls fitted with their preferred emulsified scented sleeves compared to non-scented bowls, thus highlighting that personalized enrichment strategies may be more effective than standardized approaches. Given the sustained preference observed across 14 days, scented sleeves may also serve as a form of olfactory enrichment, potentially influencing drinking behavior through affective engagement. However, the behavioral mechanisms underlying this response should be investigated in future studies. Water intake further varied by diet composition (wet food vs. dry food) and time of day. These findings warrant further investigation to evaluate whether preferred scented sleeves may offer a simple and effective hydration strategy for improving water intake in domestic dogs. Future research should investigate additional factors that may influence water consumption in pet dogs, such as seasonal changes, ambient temperature, feeding schedules and times, age, breed, prior exposure to scents, and underlying health conditions, using a larger sample size. Furthermore, future research should explore the practical implications of these findings to dogs housed in confined (e.g., shelters, laboratories) or clinical settings (e.g., during hospitalization), particularly for dogs with reduced appetite or hydration risk, such as geriatric or convalescent individuals. As this area remains underexplored, further longitudinal studies are necessary to determine if using silicone scented sleeve bowls promotes sustained increase in water consumption beyond two-week periods and whether this increase translates into measurable physiological health benefits, such as improved hydration or health markers in dogs. Additionally, similar experiments can be expanded in other companion animals like cats, who are at risk of hydration-related health issues.

## Data Availability

The datasets presented in this study can be found in online repositories. The names of the repository/repositories and accession number(s) can be found below: https://github.com/ritzp30-dot/sleeve-study.
